# CAR T-cells for pediatric solid tumors: where to go from here?

**DOI:** 10.1007/s10555-024-10214-6

**Published:** 2024-09-24

**Authors:** Tina Trautmann, Natalia Yakobian, Rosa Nguyen

**Affiliations:** Pediatric Oncology Branch, NCI, NIH, NCI, 10 Center Drive, 1W-5832, Bethesda, MD 20892 USA

**Keywords:** Solid tumors, CAR, Adoptive T-cell therapy, Pediatric oncology

## Abstract

Despite the great success that chimeric antigen receptor (CAR) T-cells have had in patients with B-cell malignancies and multiple myeloma, they continue to have limited efficacy against most solid tumors. Especially in the pediatric population, pre- and post-treatment biopsies are rarely performed due to ethical reasons, and thus, our understanding is still very limited regarding the mechanisms in the tumor microenvironment by which tumor cells exclude effectors and attract immune-suppressive cells. Nevertheless, based on the principles that are known, current T-cell engineering has leveraged some of these processes and created more potent CAR T-cells. The recent discovery of new oncofetal antigens and progress made in CAR design have expanded the potential pool of candidate antigens for therapeutic development. The most promising approaches to enhance CAR T-cells are novel CAR gating strategies, creative ways of cytokine delivery to the TME without enhancing systemic toxicity, and hijacking the chemokine axis of tumors for migratory purposes. With these new modifications, the next step in the era of CAR T-cell development will be the clinical validation of these promising preclinical findings.

## Introduction

Chimeric antigen receptor (CAR) T-cell therapy comprises genetically modified autologous T-cells that express a synthetic tumor-targeting CAR. Following lymphodepleting (LD) chemotherapy, this *ex vivo* manufactured “living drug” is reinfused into patients to eliminate cancer cells. The indications for CAR T-cells are quickly expanding and have changed clinical practice. To date, the U.S. Food and Drug Administration (FDA) has approved CD19-targeted CAR T-cells for patients with relapsed/refractory (R/R) leukemia and lymphoma, and B-cell maturation antigen (BCMA)-targeted CAR T-cells for patients with R/R multiple myeloma [1]. In a subset of these patients, CAR T-cells can induce long-term remissions and possibly cures alone or when combined with other consolidative therapies such as bone marrow transplantation [2–4]. In a study by the National Cancer Institute, close to two-thirds of the 43 patients with R/R B-cell lymphoma or chronic lymphocytic leukemia achieved a complete response (CR) after therapy with axicabtagene ciloleucel, a CD19-targeted CAR T-cell therapy. Additionally, 76% of these individuals remained in long-term remission without further therapy [5]. Comparable results were reported in a single-institution study of patients with lymphoma treated with tisagenlecleucel therapy, reporting a CR rate of 55% and 5-year event-free survival rates of 60% [6]. These studies and others have shown that durable responses were associated with a profound initial response, an absence of extramedullary disease, low tumor burden, receipt of LD chemotherapy, and high peak expansion of CAR T-cells—features that are likely shared in solid tumors as well. On the contrary, the development of CAR T-cells for solid tumors has not successfully transitioned beyond phase I/II trials due to the lack of robust activity despite promising preclinical work [7]. Many reviews have discussed potential key challenges and clinical determinants of success for CAR T-cells in solid tumors [8–16], some of which remain poorly understood, including (1) what characteristics of CAR T-cells enhance the trafficking to the tumor site, (2) what barriers in the tumor microenvironment can be therapeutically targeted to improve CAR T-cell performance, and (3) how CAR T-cells can be engineered to extend their function and activation without increasing toxicity in patients. The goal of this review is not to add another comprehensive review on this topic, but instead, to summarize new preclinical and clinical insights into factors that contribute to these challenges by highlighting key articles and concepts as well as providing suggestions on how to overcome them while balancing on-target off-tumor toxicity for the next generation of CARs.

### *CAR* T-cell efficacy in solid tumors

The clinical landscape of CAR T-cell studies in pediatric solid tumors has been predominated by disialoganglioside (GD2)-CAR T-cells [17–22] (Table 1). However, across these studies, variable responses were reported. Among the most promising studies is a recent phase I/II trial (NCT03373097) of a 3rd generation (3G) GD2-CAR (GD2-CAR01) with a rimiducid-inducible caspase 9 suicide switch. In this study, researchers demonstrated feasibility and reported no safety concerns, testing three dose levels (3, 6, and 10 × 10^6^ CAR^+^ T-cells/kg) following standard LD chemotherapy [17]. T-cells were transduced at high levels (> 70%) using a retroviral vector (iCasp9.2A.GD2.CD28.4-1BB.zeta) and expanded with IL-7 and IL-15. No dose-limiting toxicity (DLT) was reported in the 27 treated children with R/R neuroblastoma. Peak CAR T-cell expansion occurred in the second week after infusion, correlated with the dose level at which the patient was enrolled, and persisted for at least 3 months in 75% of participants. Interestingly, patients with low expansion (< 5% in the peripheral blood) had a significantly higher proportion of terminally differentiated CD45RA^+^ CCR7^−^ CD4 T-cells in their infusion product. The trial reported a 63% response rate among which there were 9 CRs (33%) and 5 of 9 sustained CRs at a median follow-up of 1.7 years. Importantly though, the nine responders had SIOPEN MIBG scores of less than 4 with four patients having a score of 0, highlighting that responses in these patients and likely others with solid tumors occur in individuals with limited disease burden and that T-cell subsets may play an important role in anti-tumor activity of CAR T-cells.

**Table 1:** CAR T-cell trials in solid tumors: a representative selection of CAR T-cell trials is shown, listing the disease, target antigens, CAR design, and outcome data. Only registered and completed trials with information on measurable disease at treatment start were included

Disease (*N*)	Target	CAR features	Best responses	NCT, REF*
NB (11 total)	GD2	3G GD2 CAR (CD3ζ, OX40, CD28, iC9)	Cohort 2–3 (CAR, CTX ± PD-1): 2/7 CR, 1 SD, 4/7 PD	NCT01822652 [18]
NB (19 total) 11 with MD	GD2	1G GD2 EBV-CTL CAR (CD3ζ)	Cohort with active disease: 3/11 CR, 2 SD, 1 PD	NCT00085930 [20, 21, 26]
NB (12)	GD2	2G GD2 1RG-CAR (CD3ζ, CD28)	0 OR	NCT02761915 [22, 27]
NB (10)	GD2	4G 4SCAR-GD2 (CD3ζ, 41BB, CD28, iC9)	4 SD	NCT02765243 [28]
NB (27)	GD2	3G GD2 CAR (CD3ζ, CD28, 41BB)	9 CR, 8 PR, 5 SD	NCT03373097 [17, 29]
OS (16), ES (1), PNET (1), DSRCT (1)	HER2	2G HER2-CAR (CD3ζ, FRP5, CD28)	4 SD (3 OS, 1 DSRCT)	NCT00902044 [30, 31]
NB (3), OS (12)	GD2	3G GD2-CAR (CD3ζ, CD28, OX40, iC9)	10 SD, 2 PD. All patients eventually progressed	NCT02107963 [19, 32]
Pediatric solid tumors (11) 10 with MD	EGFR	2G EGFR806-CAR (CD3ζ, 41BB, EGFRt)	3 mixed responses	NCT03618381 [33]
Pediatric solid tumors (16), 9 infused	B7-H3	B7H3-CAR (CD3ζ, 41BB, DHFRdm, EGFRt)	3 SD	NCT04483778 [34]
DIPG, DMB (4)	GD2	2G GD2-CAR (CD3ζ, 41BB, iC9). Note: IV, ICV	3 patients with clinical and radiographic improvement	NCT04196413 [35–37]
HCC (6)	GPC3	2G GPC3-CAR (CD3ζ, 41BB, RUNX3). Note: ± CT017 TKI	1 PR, 2 SD	NCT06198296 [38, 39]
Epithelial tumors (12)	EpCAM	EpCAM-CAR (CD3ζ, Dectin-1, CD28, or 4-1BB)	5 SD, 8 PD	NCT02915445 [40]
CRC (10)	CEA	2G CEA-CAR (CD3ζ, CD28)	7 SD	NCT02349724 [41]
HCC (14), PC (7), CRC (2)	CD133	2G CD133-CAR (CD3ζ, CD28, 41BB)	3 PR, 14 SD	NCT02541370 [42]
GBM (58)	IL-13Ra2	2G IL-13Ra2-CAR (CD3ζ, 41BB). Note: ICT, ICV, ICT/IVC	2 CR, 29 SD, 2 PR, 1 CR	NCT02208362 [43, 44]
GC (28), other (9)	CLDN 18.2	2G CLDN18.2-CAR (CD3ζ, CD28)	GC: 16 PR, 5 SD, 7 PD Other: 2 PR, 4 SD, 3 PD	NCT03874897 [45, 46] NCT04581473 [47]
CRC (9)	GCC/IM96	GCC-CAR(IM96), not specified	DCR 66.7%, ORR 11.1%	NCT05287165 [48]
TNBC (4), NSCLC (2) 5 with MD	ROR1	2G ROR1-CAR (CD3ζ, 41BB)	4 mixed responses; TNBC: 1 SD. All patients eventually progressed	NCT02706392 [49, 50]
PC (16) 14 evaluable	EGFR	2G EGFR-CAR (CD3ζ, 41BB)	8 SD, 4 PR, 2 PD	NCT01869166 [51–53]
MPM, lung cancer, breast cancer (27), 24 with MD	Meso-thelin	2G Mesothelin-CAR (CD3ζ, CD28, iC9) Note: Intrapleural application+PD-1 inhibitor (pembrolizumab)	Among 18 patients with CAR+PD-1 inhibitor: 2/18 CR, 4/18 SD	NCT02414269 [54, 55]
MCRPC (13)	PSMA	2G TGFβ-resistant PSMA-TGFβRDN-CAR (CD3ζ, 41BB, TGFβRDN)	5 SD	NCT03089203 [56]

*CR* complete response, *CRC* colorectal carcinoma, *CTX* chemotherapy, *DCR* disease control rate, *DIPG* diffuse intrinsic pontine glioma, *DMG* spinal diffuse midline glioma, *DSCRT* desmoplastic small round cell tumor, *ES* Ewing sarcoma, *GMB* glioblastoma, *GC* gastric cancer, *ICT* intratumoral, *ICV* intraventricular, *IV* intravenous, *MD* measurable disease, *MDM* malignant pleural mesothelioma, *NB* neuroblastoma, *NSCLC* non-small cell lung cancer, *ORR* objective response rate, *OS* Osteosarcoma, *PC* pancreatic cancer, *PD* progressive disease, *PNET* primitive neuroectodermal tumor, *PR* partial remission, *SD* stable disease, *TNBC* triple-negative breast cancer

In contrast to this trial, another recent study by the National Cancer Institute tested the 3G GD2-CAR, GD2.CD28.OX40.z and reported no objective responses in 12 patients with osteosarcoma and three with neuroblastoma [19]. These CAR T-cells were manufactured with retroviral vector (iC9-2A-14G2A.CD28.OX40Z), expanded in IL-2, and administered after LD chemotherapy at four different dose levels (0.1, 1, 3, and 10 × 10^6^ CAR^+^ T-cells/kg). Again, no DLT was reported. Although the peak CAR T-cell expansion was decent (range, 0.01–3.4 × 10^5^ copies/mcg of DNA), none of the 13 patients showed clinical responses (partial responses [PRs] or CRs), not even those 7 of 13 with low disease burden. However, despite the lack of activity, this study identified important factors that were associated with good CAR T-cell expansion. Patients with an abundance of naive CD45RA^+^ CCR7^+^ CD8 T-cells in their infusion product had good expansion, while patients with mainly CD45RA^+^ CCR7^−^ CD38^+^ TBET^+^ CD11b^+^ CD122^+^ terminally differentiated effector TEMRA CD8 T-cells were poor expanders. Additionally, this study established a link between CXCR3^+^ or CXCR3^hi^ classical monocytes and GD2-CAR T-cells and showed that their association can modulate CAR T-cell expansion and function.

In a third phase I clinical study (NCT04196413), pediatric and young adult patients with diffuse intrinsic pontine glioma (DIPG) and diffuse midline glioma (DMG) receive retrovirally transduced GD2-CAR T-cells (14G2A–4-1BB–CD3Ζ) in an ongoing study. These effectors were expanded in IL-7 and IL-15 with dasatinib on days 3 and 5 to improve T-cell fitness [23, 24]. Three out of four initial patients treated on the first dose level at 1 × 10^6^ CAR^+^ T-cells/kg had clinical and radiographic improvement without reported on-target off-tumor toxicity. Further interim data from this trial corroborated these early promising findings in 13 patients [25]. Although GD2-CAR T-cells were tolerated on dose level 1, three patients subsequently developed grade 4 cytokine release syndrome (CRS) at the next dose level. All individuals developed tumor inflammation–associated neurotoxicity (TIAN), which was medically managed and resolved. These results prompted the study team to amend the protocol and administer intracerebroventricular GD2 CAR T-cells without LD chemotherapy preceded by a course of intravenous GD2-CAR T-cells with LD chemotherapy. On this modified arm, all patients have tolerated the intracerebroventricular injections without reported DLTs.

Even though CAR T-cell therapy trials in adult solid tumors target a larger variety of antigens in a more heterogeneous patient population, the anti-tumor efficacy of these treatments mirrors the response rates in pediatrics (Table 1). Given that to date, CAR T-cells are feasible and generally safe in clinical trials, the presented data raises the question of whether perhaps, there should be a shift in the focus of future clinical CAR T-cell studies in solid tumors. For example, one may want to target other antigens, combine CAR T-cells with immune-modulatory treatments, and expand on more in-depth correlative analyses to gain a more nuanced biological understanding of CAR T-cell biology.

### What is an ideal CAR T-cell for solid tumors?

Based on the clinical responses from completed studies (Table 1), it is clear that CAR T-cell therapy in solid tumors has room for improvement. But what aspects of this treatment should be improved? Based on our mechanistic understanding of the challenges in the tumor microenvironment (TME) and the wealth of preclinical data on CAR engineering to enhance efficacy and T-cell fitness, we crafted an ideal CAR for solid tumors.

#### The ideal target

The problem: The “perfect” antigen is an oncogenic driver that is highly abundant and homogenously expressed on tumor cells while absent on healthy tissues and is widely shared among patients (“public epitope”) and across tumor types as a pan-marker [57, 58]. Most naturally occurring CAR targets are tumor-associated antigens (TAAs) and do not fulfill these criteria. While one can live with B-cell aplasia following CD19-CAR T-cell therapy, cross-reactivity against other TAAs can be detrimental to the patient [59, 60]. For example, 39% to 64% of patients receiving CD19-CAR T-cells develop immune effector cell–associated neurotoxicity syndrome (ICANS) that can range from mild neurologic symptoms to fatal encephalopathy [61, 62]. The occurrence of ICANS is mechanistically linked to the expression of CD19 in brain mural cells that form an integral part of the blood–brain barrier and when damaged, may allow CD19-CAR T-cells to invade the brain and cause neurotoxicity [63]. Interestingly, despite the exuberantly high expression of GD2 in mature neurons [64], patients treated with GD2-CAR T-cells do not develop neuronal cell death or as a result, overt encephalopathy, or severe peripheral neuropathy [17, 20–22, 65]. However, individuals with brain tumors but not those with extracranial solid tumors can manifest TIAN, which is thought to be caused by neuronal dysfunction due to local inflammation [66]. Clinical experience in humans is in stark contrast to preclinical findings in a study in which mice were treated with an affinity-enhanced GD2-CAR (GD2-E101K) and developed fatal encephalitis 19 to 27 days after GD2-CAR T-cell injection (though repeated studies by other groups were not able to reproduce these findings) [67, 68]. A pathologic review of the murine brains revealed T-cell infiltration and neuronal destruction. It is conceivable that the high antigen density threshold for most CARs is a possible reason why CAR T-cells do not cause more toxicity, considering that there are normal vital organs that share antigens with tumors such as the brain [22, 69]. Such toxicity may occur if this antigen threshold is lowered as in affinity-enhanced CARs. Other factors that mitigate toxicity remain to be discovered.

Strategies: The accurate identification of antigens for CAR therapy is essential. Especially in solid tumors, there is a scarcity of tumor-specific or tumor-associated targets not expressed in vital organs and tissues. Although tumor tissues are required for target discovery, we live in an era in medicine and science where there are large datasets publicly available that allow for *in silico* discovery work. Advances in bioinformatics and the broader availability of biocomputational resources have allowed for a comprehensive yet more nuanced re-analysis of these datasets. For example, recently, over 1500 RNA data sets from the St. Jude Cloud and the NCI TARGET cohort were analyzed for cancer-specific exons that present promising targets due to their exclusivity in cancer [70]. After cross-referencing potential targets with expression in normal tissues using the Genotype-Tissue Expression (GTEx) consortium data, the authors found 37 new gene-level or alternatively spliced exon targets that encode surface or matrix proteins and are absent in normal tissue. Approaches like this are particularly important in pediatric cancer where re-biopsies are rarely indicated for clinical reasons and are difficult to ethically justify for research purposes.

Antigens of recent interest are oncofetal proteins that are highly expressed during development but absent in most healthy tissues after birth, rendering them an attractive option for CAR development [71]. In addition, some antigens may have several isoforms, some of which are tumor-restricted, which may further minimize on-target off-tumor cross-reactivity and increase the therapeutic window of CAR T-cells [72, 73]. Ideally, target candidates also serve as oncogenic drivers for tumors. In this capacity, downregulation of the antigen and immune evasion are countered due to the antigen’s essential role in tumor growth [72, 73]. Glypican-2 (GPC2), a member of the glypican family, possesses most of these features. GPC2 is a neurodevelopmental heparan sulfate proteoglycan and is silenced after birth except for continued expression in the male testis [74]. The isoform GPC2-201 is expressed by several pediatric solid tumors including embryonal brain tumors and neuroblastoma [69, 72, 74–77]. In neuroblastoma, GPC2 has pro-tumorigenic effects by signaling through the WNT/β-catenin pathway. Preclinical efforts have identified efficacious CARs targeting GPC2 that are in clinical development across different centers in the U.S. Promising oncofetal antigens for CAR T-cells in adult cancers include the carcinoembryonic antigen (CEA) (NCT02349724), claudin 6 (CLDN6) (NCT04503278), or tumor-associated glycoprotein 72 (NCT05225363). Results from these clinical trials are pending.

#### The ideal CAR

The problem: The ideal CAR can sense specific antigens at a wide range of densities, potently activate T-cells to kill targets, and persist on the cell surface without causing tonic signaling. In comparison to TCRs, CARs typically require a much higher antigen density (> 1000 molecules/cell) due to factors like limited kinase recruitment, underdeveloped immune synapses, decreased co-receptor engagement, and increased activation of downstream regulators [9]. Though antigen specificity is a desired feature in CARs, we hypothesize that low level of antigen exposure may be beneficial for CAR persistence and long-term function. Thus, similar to the Goldilocks principle, CAR design has to enable “just the right amount” of signaling to activate CAR T-cells and trigger killing without causing exhaustion over time.

Strategies: In CARs that target highly expressed antigens, like GD2, CAR engineering becomes a central tool to fine-tune downstream signaling and CAR function. For example, the single-chain variable fragments of a 14G2a-based GD2-CAR exhibit antigen-independent clustering that causes tonic signaling and early exhaustion [78]. This phenomenon was not found with a CD19-CAR with structural similarities in the CAR construct. However, by using a CD28 instead of a 4-1BB costimulatory domain, CAR clustering and tonic signaling were dramatically reduced and CAR function improved. CARs that target low-density antigens like the GPC2-CAR also benefit from CAR engineering. Here, the choice of a CD28 transmembrane domain optimally enhances downstream signaling and CAR efficacy [69, 76].

**Fig. 1:**
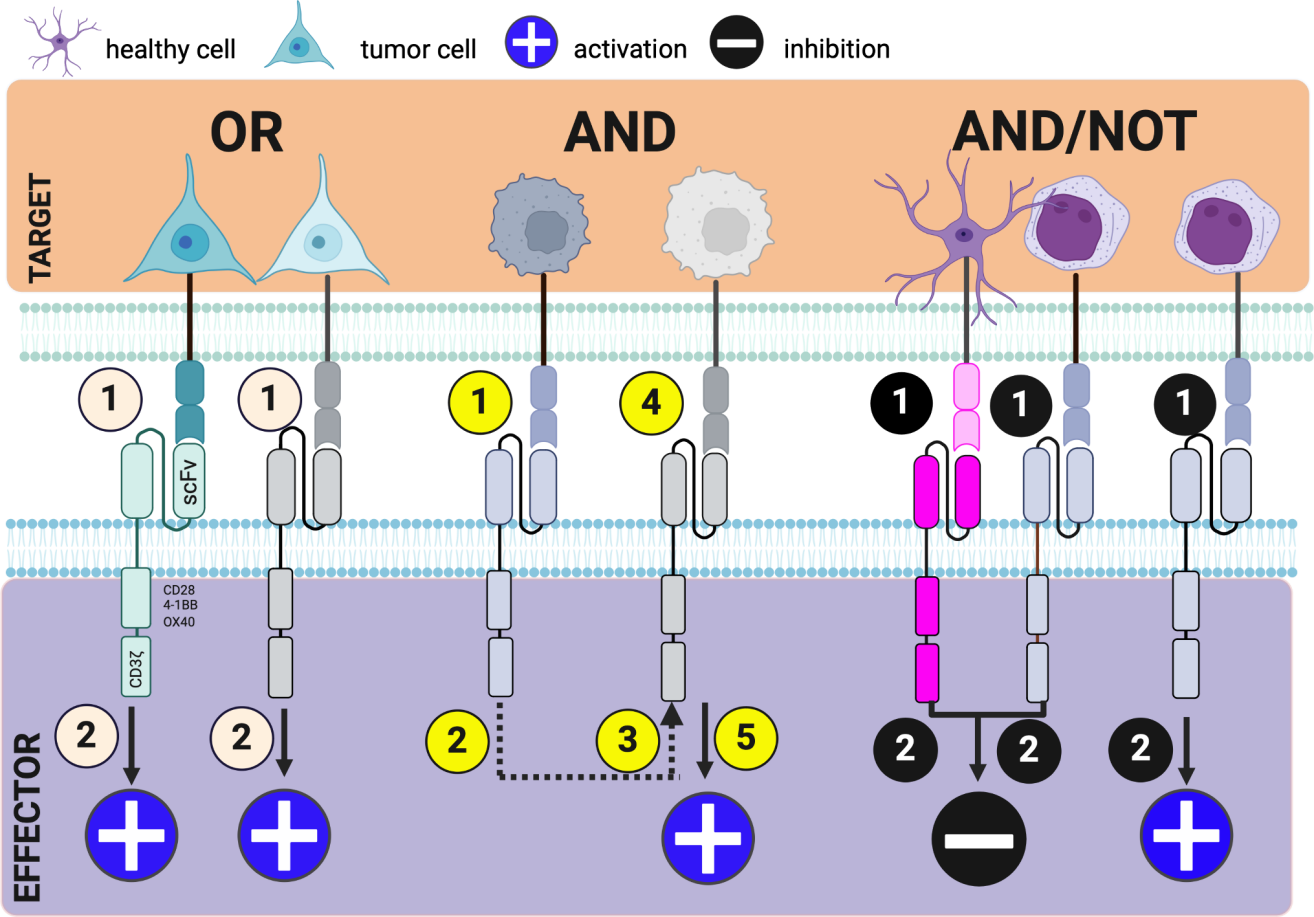
Summary of logic gates. OR gating (e.g., bispecific CAR): CAR is activated in response to a cell expressing antigen A or B. AND gating (e.g., SynNotch): SynNotch receptor releases a transcription factor upon activation that translocates into the nucleus and turns on the expression of a CAR for antigen B. AND/NOT (e.g., inhibitory (i) CAR): iCAR dampens the T-cell response when encountering an antigen on healthy tissue

Other CAR engineering efforts have focused on Boolean logic–gating strategies to improve the specificity. Because combinatorial antigen sensing is required for CAR activation in this system, in theory, these receptors can better discriminate between tumor and healthy tissue, thereby expanding the repertoire of potential antigens (Fig. 1). The synNotch IF–THEN circuit incorporates a synthetic notch receptor, which upon antigen-specific engagement triggers the transcription of a conventional CAR against a TAA [79–84]. Thus, while both antigens are tumor-associated, their joint presence is required for CAR activation and cytotoxicity. However, despite effectively discriminating between single- and dual-antigen tumors, co-localization of TAA in healthy tissues remains a problem. Furthermore, this system can lead to sustained CAR expression and potential off-target effects as T-cells migrate into healthy tissue. Moreover, although the synNotch system has shown promising results in preclinical studies of neuroblastoma using GD2 and B7-H3 CARs, the scalability of this system poses a significant hurdle for clinical translation [81]. In the logic-gated intracellular network (“LINK”) AND gate platform, two proximal TCR signaling molecules, LAT and SLP76, are fused to membrane-bound scFvs and co-localize upon concurrent binding to induce T-cell activation [85]. Unlike in the synNotch system, this design enables reversible activation, contingent upon continuous and proximate interaction of both TAAs. In a preclinical ROR1 toxicity model, LINK CAR T-cells eradicated tumors presenting both antigens without causing toxicity. Mice treated with synNotch-engineered T-cells died from on-target off-tumor toxicity in this model [84]. CARs with “AND NOT” gating co-express a prototype CAR in *trans* with an inhibitory CAR (iCAR) [86]. The former triggers T-cell activation upon binding of a TAA, while the latter dampens activation when encountering an antigen on healthy tissue. The antigen-binding domain of iCARs is commonly fused to the signaling domains of negative checkpoints, such as PD-1 and CTLA-4 [87]. Temporary inhibition of cytokine secretion, cytotoxicity, and proliferation can provide rest to the T-cells and reinvigorate their function in the long term. These approaches demonstrate that re-purposing the TCR signaling machinery could open novel avenues for CAR engineering.

Although combinatorial antigen-sensing may improve therapeutic safety, antigen escape remains an important mechanism of immune evasion and therapy failure that needs to be addressed. Bi-specific CARs target two antigens or two epitopes of one antigen (OR gate) [77, 88]. This can be achieved through the administration of a mixed cell product or a bicistronic or bivalent CAR [9]. As opposed to AND gates, this method allows for the recognition of two TAA but does not require concurrent antigen binding or simultaneous antigen expression. In the clinic, ciltacabtagene autoleucel, a bi-specific BCMA-targeting CAR, is highly effective in R/R multiple myeloma and received by the FDA based on the results from a phase II trial that demonstrated an overall response rate of 73% and a median progression-free survival of 8.8 months [89]. Preclinical work with a bicistronic GPC2 and B7-H3-targeted CAR shows promising results in preclinical models of neuroblastoma [77]. Future studies are needed to translate this and similar efforts into the clinic.

Antigen loss or downregulation is a well-established mechanism of immunotherapy resistance [90–93]. Multi-specific CAR T-cells could be a solution to overcome this therapy resistance mechanism. For example, in a glioblastoma model, bispecific CAR T-cells targeting both interleukin-13 receptor subunit alpha-2 (IL-13Rα2) and ephrin type-A receptor 2 (EphA2) demonstrated enhanced efficacy and reduced antigen escape compared to single CAR bearing T-cells [94]. However, increasing the therapeutic pressure in this manner can result in the downregulation of multiple antigens, as observed in pediatric ALL clinical trials where some patients relapsed with CD19^low^CD22^low^ tumors after treatment with bicistronic CD19/CD22 CAR T-cells [95]. In this instance, the development of a universal or adapter CAR offers greater flexibility. These CAR T-cells are engineered with a universal receptor that can be activated by a soluble adapter molecule containing an scFv region specific to a tumor antigen [96]. This design allows for the redirection of CAR T-cells to virtually any antigen target without needing further genetic modification or new T-cell production. When a tumor downregulates a specific antigen, a new adapter molecule can be generated to stimulate CAR T-cells *in vivo*. However, this strategy still relies on the identification of tumor-specific antigens. A potentially simpler approach involves creating T-cells that target synthetic markers. Recently, Vincent et al. developed an innovative system where tumor-colonizing probiotic bacteria are engineered to produce synthetic markers that label tumor tissue [97]. Probiotic CAR T-cells (ProCARs) are then designed to target these markers, leading to antigen-independent, tumor-specific cell lysis.

#### The ideal migratory and survival skills of a CAR T-cell

The problem: One of the greatest hurdles to effective CAR T-cell therapy against solid tumors is the presence of an immune-hostile TME. Unlike in hematological cancers, the solid tumor TME is a dynamic system composed of tumor cells, immune cells, stroma cells, abnormal vasculature, and dense disorganized extracellular matrices (ECMs) all designed to facilitate tumor survival, growth, and metastasis [98–100]. Tumor cells secrete chemokines that preferentially attract pro-tumor myeloid-derived suppressor cells (MDSCs), M2 tumor–associated macrophages (TAMs), and regulatory T-cells (Tregs) [101]. Once attracted, these immune cells secrete type II cytokines (e.g., IL-4, IL-10, and IL-13) that perpetuate further recruitment of immune-suppressive cells [101, 102], induce effector exhaustion through inhibitory checkpoint ligands, and force the development of a nutrient-deplete TME [102]. For example, MDSCs and M2 TAMs secrete reactive oxygen and nitrogen species and actively consume arginine. Tregs can act as cytokine “sinks,” depleting cytokines like IL-2 [103–105]. While immunosuppressive cells thrive in this environment, these features present physical and chemical barriers to the trafficking, infiltration, and persistence of CAR T-cells, limiting their therapeutic efficacy [106].

Strategy: CAR T-cells should possess the ability to extravasate and migrate into the tumor tissue going against a less favorable oxygen and metabolic gradient. As these cells arrive at the core of solid tumors, they must have the ability to survive, kill, and expand. This can be achieved by strong activation signals, for example, through the activation of the CAR, and by receiving additional signals through chemokines, cytokines, or other nurturing molecules or cues from the environment or the effectors themselves.

T-cells can transmigrate from the bloodstream when attracted by chemokines like CXCL9/10/11 and CCL5. After transversing across stromal cells and ECM, CAR T-cells then can seek direct tumor contact via intracellular adhesion molecule-1 (ICAM1) and execute anti-tumor cytotoxicity [107]. Importantly, although tumor cells secrete chemokines like CXCL1/2, CCL2, and IL-8 that attract myeloid cells to the tumor microenvironment (TME), they typically do not secrete chemokines for which CAR T-cells express receptors [108]. In addition, manufacturing procedures aim at generating CAR T-cells with a CCR7^high^ CD62L^high^ CD45RO^+^ central memory effector phenotype because these cells exhibit greater cytotoxic capacity and lower propensity for functional exhaustion [109]. However, CCR7 and CD62L are known molecules that promote migration to secondary lymphoid tissues, contributing to a CAR T-cell-devoid TME [110–112]. Intraventricular injections in patients with brain tumors or intra-tumoral injections in breast cancers without preceding LD chemotherapy have shown to be feasible [113, 114]. Whether these alternate routes of administration improve CAR T-cell persistence in the TME and enhance cytotoxicity and clinical outcomes remains to be determined.

The persistence of CAR T-cells is measured in the peripheral blood in most clinical studies. CAR T-cell numbers are highest within the first 2 weeks after infusion and last for about a month before contracting [112]. The meaning of this information is unclear as the presence and persistence in the TME may be of greater biological importance. Although one may assume that the presence of peripheral blood CAR T-cells indirectly reflects CAR function, their presence is not a reliable biomarker for clinical responses. The best examples are the GD2-CAR T-cell studies where patients have high persistent levels of CAR (up to 3.4 × 10^5^ copies/mcg of DNA) but do not show a clinical response after therapy. One major limitation of studying the migration and persistence of CAR T-cells in solid tumors is the lack of non-invasive detection methods. CAR T-cell imaging techniques are actively being developed for this purpose. Direct radiolabeling techniques employ nanoparticles or small molecules like ^89^Zr-oxine, ^111^In-oxine, or Technetium-99 m (99mTc)-hexamethylpropylene amine oxime and render T-cells detectable by single-photon emission computed tomography (SPECT) or positron emission tomography (PET). The half-lives of these molecules are relatively short, precluding longitudinal imaging [115, 116]. Reporter gene–based labeling with herpes simplex virus 1 thymidine kinase (HSV1-tk), norepinephrine transporter, and sodiumiodide symporter has become more popular as this approach allows for longer term tracking of T-cells [117, 118]. While promising, further refinement and validation of these methods are needed to reduce imaging background, increase resolution, and confirm their prognostic value.

All effectors, including therapeutic CAR T-cells, require a functional vascular network to enter the tumor [119, 120]. However, the TME of many solid tumors propagates the development of abnormal vessels, leading to poor CAR T-cell extravasation into tumors. This occurs in a forward propagating loop. The poor vasculature leads to hypoxia, which stimulates cells in the TME to secrete large amounts of angiogenic factors including vascular endothelial growth factor (VEGF), fibroblast growth factor (FGF), transforming growth factor-β (TGF-β), and platelet-derived growth factor (PDGF) [121–123]. This in turn leads to the rapid formation of dysfunctional and distorted blood vessels, ultimately leading to more hypoxia [121, 124, 125]. These aberrant vessels have reduced expression of adhesion molecules including vascular cell adhesion molecule-1 (VCAM1) and ICAM1, hindering extravasation, and leading to poor infiltration of CAR T-cells [126–128]. Several studies have found that blocking VEGF signaling can lead to the normalization of tumor vessels and improved tracking of adoptively transferred effectors cells into the TME [129–131]. The utility of VEGF-neutralizing antibodies could enhance the migration and improve the efficacy of CAR T-cells. As a proof of principle, in a recent syngeneic mouse model of glioblastoma, the co-administration of anti-VEGF and EGFR-vIII CAR T-cells therapy improved infiltration of CAR T-cells and prolonged survival of mice compared to CAR T-cells monotherapy [132].

### What strategies will lead us to an ideal CAR T-cell therapy?

**Fig. 2:**
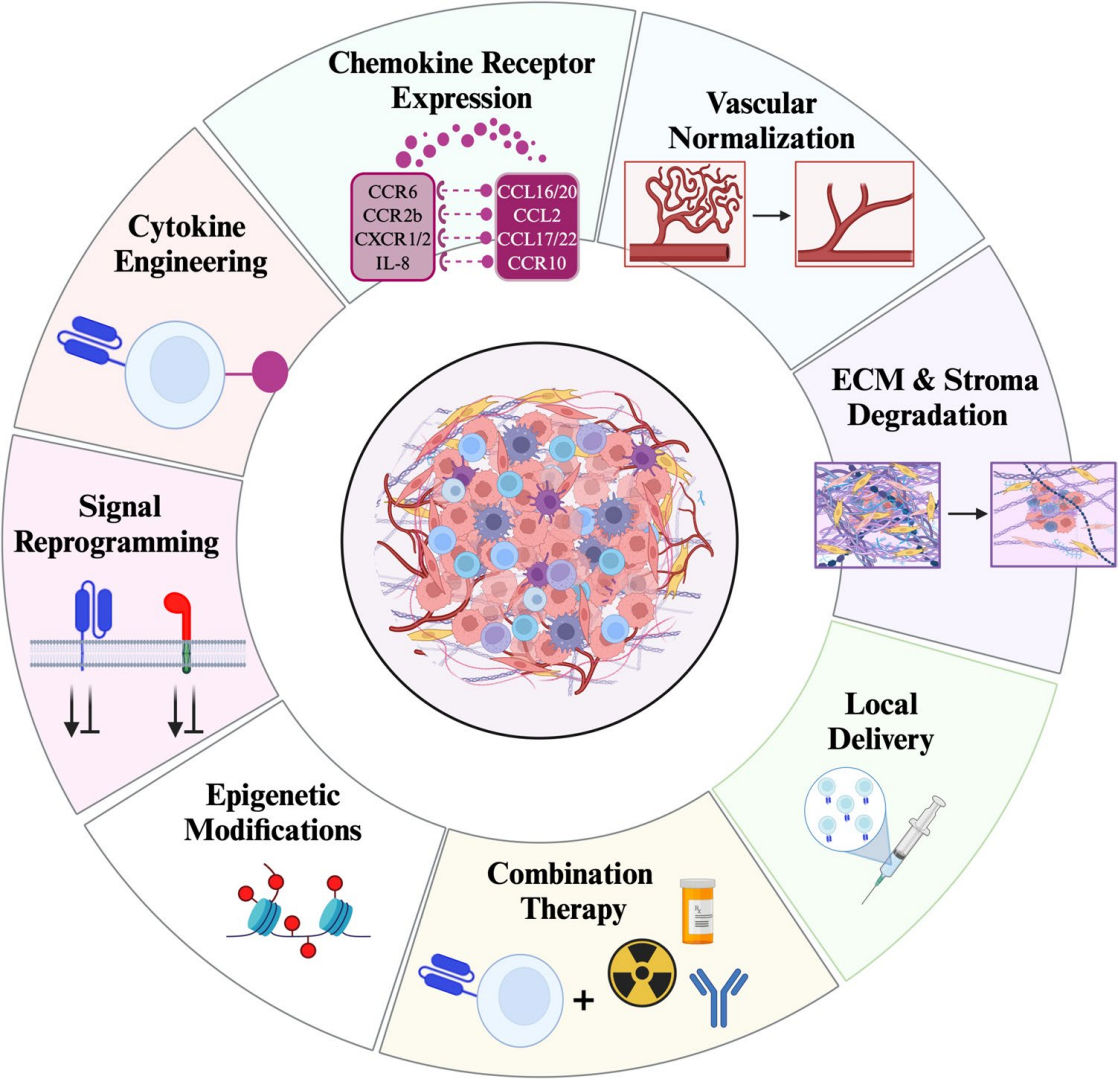
Strategies to enhance CAR T-cell therapy against the immunosuppressive TME of solid tumors. Strategies are shown to enhance CAR T-cell function, persistence, and migration. Many of these advances offer overlapping benefits, optimizing multiple aspects of CAR T-cell functionality simultaneously

In the following, we will highlight and suggest different strategies to enhance CAR T-cell therapy in solid tumors (Fig. 2). Though each of these approaches is compelling, in the end, we suspect that a combination of several strategies is needed to optimize CAR T-cells and improve clinical outcomes.

#### Combinatorial therapies

One-on-one, CAR T-cells are very potent killers of tumor cells, but they require help when facing additional challenges in the TME. As with conventional anti-cancer therapy, combinatorial therapies with CAR T-cells may have a synergistic effect and break down some of these barriers. However, since CAR T-cells are “living drugs,” one is limited by what agents to use concurrently. Lymphocytes, especially when activated, are exclusively sensitive to radiation and chemotherapy [133, 134]. In this context, LD chemotherapy is an important but undervalued aspect of treatment. Since LD chemotherapy and adoptive cell infusion occur in a timely staggered manner, CAR T-cells are typically not affected by LD chemotherapy, which is used to eradicate endogenous lymphocytes, create a niche for adoptive cells to engraft, and eliminate immune-suppressive cells in the TME. Fludarabine and cyclophosphamide (flu/cy) are the standard LD agents in leukemia and were adopted in solid tumor cell therapy. However, it is unclear whether this combination is the best for solid tumors or if patients could benefit from a third agent or a therapy modality that remodels the TME and skews the immune repertoire toward effectors. Tumor-targeted radiation could be one such modality. Radiation can eliminate tumor cells and activate the immune system in several ways such as through the cGAS/STING pathway via DNA damage or by inducing an abscopal effect or antigen spread [135–137]. Radiopharmaceutical therapy is an attractive approach to reach multiple tumor sites at the same time while administering low-dose radiation [138]. This approach was tested in immunologically “cold” tumors using ^86^Y-NM600, an alkylphosphocholine analog that accumulates in most tumor types, combined with immune checkpoint inhibitor (ICI) therapy [139]. Combination therapy led to CRs in 45 to 66% of mice compared to 0% with monotherapy and was dependent on STING expression in tumor cells. Interestingly, ^86^Y-NM600 promoted tumor infiltration by CD8 T-cells and enrichment of the T-cell memory compartment.

In another noteworthy paper, the deletion of the DNA methyltransferase 3 alpha (DNMT3A) in EphA2-CAR T-cells rendered these cells more potent against locoregional osteosarcoma [140]. The CAR T-cells had universally preserved proliferation and function despite being chronically exposed to antigen. These effects were mechanistically linked to an up-regulation of IL-10. Thus, 5′-azacytidine or decitabine could be promising combinatorial agents for CAR T-cell therapy while simultaneously epigenetically editing the tumor and immune landscape in the TME [141, 142].

#### Signal reprograming techniques

Based on promising preclinical results of CAR T-cell combination therapy with dual ICIs such as anti-PD1 and anti-T-cell immunoreceptor with Ig and ITIM domains (TIGIT), researchers have recently begun genetically engineering CAR T-cells with immunosuppressive countermeasures [143, 144]. One approach to this has been the development of chimeric immune–checkpoint switch receptors (CISR). CISR comprise a chimeric ectodomain that recognizes exhaustion signals such as PD-L1 and CD155 with an intracellular domain designed to stimulate cell activation and survival pathways such as CD28 [145]. In this way, these cells are co-opting immunosuppressive signals for the benefit of growth and survival. This model has shown success in preclinical syngeneic and xenograft mouse models exhibiting improved CAR T-cell tumor infiltration, proliferation, efficacy, and persistence [145]. Since the TME promotes high levels of these immunosuppressive factors, a strong CAR T-cell activation will preferentially occur in the tumor. However, given the robust presence of PD-L1 in the TME, these cells may get overactivation leading to systemic toxicities and premature CAR T-cell senescence. Similarly, inverted cytokine receptors (ICRs) link the extracellular domain of the IL-4 receptor to the transmembrane and intracellular domains of IL-10 [146]. In xenograft mouse models of pancreatic tumors, these CAR T-cells with ICRs demonstrated improved cytotoxicity, effector function, and cytokine release as well as a preserved less differentiated phenotype [146]. CISRs and ICRs demonstrate exciting initial results. Further studies are needed to validate these findings.

#### Cytokine/chemokine engineering

We have discussed that the chemokine repertoire in the TME attracts immune-suppressive cells rather than effector T-cells. To overcome this hurdle, CAR T-cells have been engineered to express receptors for such chemokines, engendering the ability for them to home to the TME. The list of potential candidates is long [108, 147, 148]. For example, in a xenograft model of lung adenocarcinoma, CAR T-cells with transgenic CCR6, the receptor for the chemokine CCL20, enhanced tumor infiltration and led to prolonged survival of treated mice [149]. Similar effects were noted with the transgenic expression of CCR2b, the receptor for the myeloid attractant CCL2, in a variety of tumor models [150, 151], including neuroblastoma [152]. This approach is in clinical testing in patients with R/R Hodgkin lymphoma and cutaneous T-cell lymphoma receiving CD30 CAR T-cells with transgenic CCR4 (NCT03602157), a chemokine receptor that is classically found in Tregs and type 2 T helper cells but not in effector T-cells. Preliminary results of this trial are promising with no DLT to date and CR rates of 75% [153].

CAR T-cells can also be used as a vehicle for cytokine delivery. Popular cytokines for this type of engineering are γc family cytokines, like IL-2, IL-15, and IL-21. The use of membrane-anchored IL-15 and IL-21 enhanced the cytotoxicity of GD2-CAR and E7-TCR T-cells in preclinical models of neuroblastoma and cervical cancer and limited the occurrence of NK-like T-cells after chronic antigen exposure [154]. Other cytokines that have been used for cytokine engineering are IL-12 and IL-18 [155–157]. Importantly, membrane-anchored cytokines can activate the carrier via *cis* presentation but may be able to influence bystander immune cells via transactivation. While soluble cytokines can achieve a similar effect, the obvious advantage of a cell-restricted delivery method is the local accumulation of the cytokines and potentially fewer side effects. Another elegant method of TME-targeted cytokine delivery is the use IL-12 genetically engineered myeloid cells [158]. After homing to the tumor, they remodel the immune-suppressive TME and decrease metastasis while augmenting T and NK cell activity. Critically, IL12-engineered myeloid cells had limited persistence over time, albeit acutely elevated levels in the lungs. Elevated IL-12 levels were also found in tumors. These engineered cells would be a very attractive approach as a combinatorial strategy with CAR T-cells, given that IL-12 is a potent T-cell pro-inflammatory activator [159–162]. It remains to be determined if this cell product will cause respiratory toxicity and whether it can synergize with CAR T-cells to fight cancer cells.

#### ECM remodeling approaches

As one of the hallmarks of cancer, cancer-associated fibrosis results from an excess in collagen and ECM and is a controversial topic since this process can be both pro and anti-cancer [163]. The main cells that contribute to fibrosis are fibroblasts, fibrocytes, stellate cells, and mesenchymal stem cells [164–166]. These cells exist in the body for physiologic processes like wound healing and inflammation, but in cancer, they are hijacked and reprogrammed to support the TME [163]. Several studies explored strategies to degrade the ECM in solid tumors and allow for CAR T-cells to migrate into the TME [167]. For example, hyaluronidase-producing CAR T-cells enzymatically degrade hyaluronic acid (HA), a component of the ECM implicated in tumor rigidity and density. When co-administered with ICIs, CAR T-cells with hyaluronidase enriched in the TME and exhibited better antitumor activity than without hyaluronidase in syngeneic models of lymphoma and colon cancer [168]. Generally, each solid tumor has a different composition of the ECM; thus, strategic efforts should aim at broadly characterizing these components to attempt a concerted effort to target them. Attempts to deplete ECM-producing cells like FAP-expressing fibroblasts can be successfully undertaken with antibody–drug conjugates or CAR T-cells [169–171]. These and related strategies are still in preclinical development.

Lastly, the area of tumor-mediated post-translational modifications has gained more attention in the past years. Altered glycosylation as a characteristic of carcinogenesis frequently manifests as incomplete synthesis of O-glycans and increased branching of N-glycans [128, 172]. Whereas the former provides neoantigens for cellular therapies, N-glycans may directly impact tumor cell recognition or conceal epitopes. In this role, N-glycans protect solid tumors from synapse formation and ultimately, CAR T-cell killing. Inhibition of N-glycan synthesis with the glucose/mannose analog, 2DG, could offset this shield and restore susceptibility to CAR T-cells [173].

#### Logistical approaches

The manufacturing time for CAR T-cells presents an additional challenge to delivering cell therapy timely, particularly in patients with rapidly progressing cancer. The manufacturing process typically involves leukapheresis, T-cell isolation and activation, CAR transduction, and *ex vivo* expansion [174]. The average vein-to-vein time, including transportation, ranges from 3 to 5 weeks, often necessitating bridging therapy [175]. It is therefore critical to accelerate CAR T-cell production if possible. Tackling this challenge, next-generation manufacturing protocols try to accelerate CAR T-cell production through *in vivo* or *in situ* transduction methods. For example, *in vivo* generation of CAR T-cell uses lentiviruses, adeno-associated viruses, lipid nanoparticles, and polymeric nanocarriers, with targeting achieved via single-chain variable fragments for CD4, CD8, or CD3 [176–179]. Pandit et al. developed the Drydux technology to produce CAR T-cells *in situ* within 3 days post T-cell isolation using a macroporous biomaterial scaffold [180]. Preclinically, this approach successfully generated CAR T-cells and induced long-term remissions in preclinical models of ovarian, lung, and pancreatic cancer. Although further biomechanical studies are needed to fully delineate the properties of Drydux as an *in situ* transduction vehicle, early results demonstrate a promising technology for accelerating turnaround times. With all the above-mentioned approaches, risks of off-target gene delivery and concerns for genotoxicity necessitate further research to ensure human safety and efficacy. Another process of accelerating CAR T-cell manufacturing is the generation of CAR T-cells without *ex vivo* activation and expansion. This process can generate CAR T-cells ready for infusion within 24 h of T-cell isolation [181, 182], bypassing the expansion stage and preserving T-cell stemness, which is linked to prolonged therapeutic benefit [183]. In a phase I trial for multiple myeloma, patients treated with 24-h manufactured anti-CD19 CAR T-cells tolerated the infusion and showed a 98% overall response rate, with CAR T-cells detectable in 71% of patients at 12 months [181]. Potential concerns regarding CAR detection and meeting the regulatory requirements on product release remain a challenge with this approach. Ongoing trials are needed to further clarify the safety and efficacy of these products, emphasizing the importance of understanding biological differences across manufacturing platforms.

## Conclusions

In reviewing the latest literature on CAR T-cell development, we think that there is momentum in the field to improve this treatment for patients with solid tumors. Though still in the early stages of development, recent years have shown that new creative approaches, advances in technology, and an increasing number of clinical trials have allowed us to understand and tackle the most challenging aspects of CAR T-cell therapy in solid tumors. While these scientific advances are ongoing, the mindset in clinical trials must change with regard to correlative biospecimens. They are the most valuable source for researchers and clinicians to learn what CAR, host, and tumor-related factors prohibit or support CAR T-cell therapy and will allow us to determine in what direction we need to go from here on.

## Data Availability

No datasets were generated or analysed during the current study.
